# Society Meetings

**Published:** 1914-07

**Authors:** 


### SOCIETY MEETINGS

---

Brief reports and announcements of meetings of societies of Western New York, and those of general scope, are requested from Secretaries. Copy should be on hand the fifteenth of the month. Full transactions will be published at cost of composition.

---

**Buffalo Academy of Medicine,** Annual Meeting, at Academy rooms, Tuesday evening, June 9, 1914. Officers elected:

President—Dr. John H. Pryor. Retiring president, D. H. R. Frick.

Treasurer—Dr. Lawrence Hendee. Re-elected.

Trustee for 3 years—Earl P. Lathrop.

Section officers:

Section of Medicine:
Dr. Theodore M. Leonard, chairman.
Dr. Carl Leo-Wolf, secretary.

Section of Surgery:
Dr. Charles W. Bethune, chairman.
Dr. Prescott LeBreton, secretary.

Section of Obstetrics and Gynocology.
Dr. James E. King, chairman.
Dr. Charles Banta, secretary.

Section of Pathology:
Dr. B. F. Schreiner, chairman.
Dr. John L. Eckel, secretary.

The Report of the Secretary was read by Dr. Herbert A. Smith.

Thirty-two regular meetings and one special meeting were held, with an average attendance of 76.

Twelve council meetings during the year.

The total number of members in good standing, June 9, 1914, was 351, a net gain of 22 members.

The Treasurer's report was very gratifying, showing an excellent financial condition.

The retiring President, Dr. Harry R. Frick, reviewed the work of the organization since its conception, its steady growth and future possibilities, emphasizing the need for an academy building.

**The American Society for Physicians' Study Travels** has abandoned its proposed trip for this year, on account of preparations for a tour around the world next year.

---

**The American Association of Medical Milk Commissions** held its eighth annual meeting, June 19 and 20. The first morning session, devoted to business and the afternoon session, to scientific papers, were held at the Hotel Seneca. After an automobile ride, further papers were presented at the Oak Hill Country Club after dinner. After the second morning session and luncheon, the members went to Avon by trolley, visited the Elm Place Farm, where more papers were read and the meeting adjourned after dinner at the Avon Inn.

---

**The Elmira Academy of Medicine** at its meeting of June 3, presented the following program: Sacro-iliac Sciatica, Dr. N. H. Sobel; Echin acea, Dr. Mary Potts.

---

**The Clinical Congress of Surgeons of North America** will hold its fifth annual session in London, Eng., during the week of July 27. A long program of papers and clinics by eminent surgeons, including surgical specialists, has been arranged.

---

**National Dental Association, Rochester, N. Y.,** Opening General Session, Tuesday, July 7th, 11 A. M., Exposition Park.

Address of Welcome:

Governor Martin H. Glynn ............. Albany, N. Y.
Mayor Hiram H. Edgerton ........... Rochester, N. Y.
Roland B. Woodward, on behalf of the Chamber of Commerce ....................... Rochester, N. Y.

Response:

Dr. B. Holly Smith ..................... Baltimore, Md.

President's Address:

Dr. Homer C. Brown .................. Columbus, Ohio

Oration: "The Functions of Dentistry and Medicine in Race Betterment." By Dr. Victor C. Vaughn, (M. D.) ......................... Ann Arbor, Mich.

Tuesday, 8 P. M., Reports of Researches of the Scientific Foundation and Research Commission.

"Studies on the Relation of Systematic Infections to Dental Foci." By Dr. Thomas B. Hartzell.......... Minneapolis, Minn.

"Studies on Salivary Analysis." By Dr. Russell W. Bunting .............................. Ann Arbor, Mich.

"Studies on the Formation of Calculus." By Dr. G. V. Black ................................ Chicago, Ill.

"Studies on the Dental Pulp." By Dr. Frederick B. Noyes .................................. Chicago, Ill.

(a) "Metallurgical, Electro-Chemical and Physical Studies."
(b) "Metabolism of Tricalcic Salts." By Dr. Weston A. Price ............................Cleveland, Ohio

July 8th, Wednesday, 8 P. M.

"The Early Recognition of Pre-cancerous Lesions of the Mouth and Tongue." By Dr. Joseph C. Bloodgood, (M. D.) ..........................Baltimore, Md.

"Oral Manifestations in Syphilis." By Grover W. Wende (M. D.) ..............................Buffalo, N. Y.

Dr. Gaylord, Buffalo, and Dr. Plumley, Rochester, are Medical discussors on the Drs. Bloodgood and Wende papers. In addition to the above a very full program will be presented by members of the Dental profession and many of these papers no doubt will be interesting to the Medical men.

A Cordial invitation is extended to all reputable practitioners of Medicine.

Homer C. Brown, President,
Columbus, Ohio
Otto U. King, Secretary,
Huntington, Ind.

---

**The Lake Keuka Medical and Surgical Association** will meet this year at Grove Springs Hotel, near Hammondsport, on July 9th and 10th. Papers will be read by prominent physicians from Buffalo, Rochester, Syracuse, Elmira, New York City, Philadelphia and Albany, a very interesting and instructive program has been arranged. All the physicians in the state are invited and all residing in Erie, Allegany, Chemung, Livingston, Monroe, Onondaga, Ontario, Schuyler, Seneca, Steuben, Tioga, Wayne, Wyoming, and Yates counties are eligible for membership on the payment of one dollar dues.

The officers for this year are: President, Floyd S. Crego, M. D., Buffalo; vice-president, A. J. Knickerbocker, M. D., Geneva; Secretary, E. C. Foster, Penn Yan, N. Y.

Physicians who go by train should take the D., L. & W. train from Buffalo leaving here at 9.30 A. M., Wednesday or Thursday A. M. to Bath and Hammondsport. Those who go by auto, via Avon, Canandaigua and Penn Yan.

---

**Medical Society of the County of Erie**, met in Alumni Hall at the University of Buffalo on Monday evening, June 15, 1914, the President, Dr. John B. Woodruff, presiding.

The minutes of the previous meeting were approved as published and the minutes of the Council held June 1st were read and adopted.

Dr. Wende, Chairman of the Membership Committee presented the application of Dr. Joseph C. Wallace, who was thereupon elected to membership. He also presented the names of Dr. Bourne, of Hamburg, Dr. Trull, of Williamsville, and Dr. B. Lothrop, of Buffalo, for recommendation to the State Society to be placed on the retired list.

Dr. Bonnar made a verbal report of the work of the Censors and said that they had collected fifty dollars fine from one conviction.

Dr. Lytle reported for the Survey Committee stating that out of 585 printed requests sent out to members only 191 had been returned with the information added.

Treasurer Lytle reported 83 names of physicians who were in arrears on June 1st with their dues. He said that the Council of the State Society had adopted a ruling that all members in arrears with their dues on June 1st would not be entitled to mal-practice defense, nor the Bulletin, nor the Directory. On his motion all members received at the October and December meetings of this year will be entitled to free membership for the balance of 1914.

Papers were read by Dr. L. B. Dorr on the Action of Sulphate on Hardening of the Arteries; by Dr. H. W. Cowper on Eye in General Practice; and by Dr. J. H. Dowd on Facts, Fancies and Failures.

After a discussion of the papers the Society adjourned.

